# Association of low-dose glucocorticoid use and infection occurrence in systemic lupus erythematosus patients: a prospective cohort study

**DOI:** 10.1186/s13075-022-02869-9

**Published:** 2022-07-28

**Authors:** Kazuya Abe, Yuichi Ishikawa, Yasuhiko Kita, Nobuyuki Yajima, Eisuke Inoue, Ken-ei Sada, Yoshia Miyawaki, Ryusuke Yoshimi, Yasuhiro Shimojima, Shigeru Ohno, Hiroshi Kajiyama, Kunihiro Ichinose, Shuzo Sato, Michio Fujiwara

**Affiliations:** 1 Department of Rheumatology, Yokohama Rosai Hospital, 3211, Kozukue-cho, Kohoku-ku, Yokohama, Kanagawa Japan; 2 Department of Allergy and Clinical Immunology, Chiba University Hospital, Chiba, Japan; 3 The First Department of Internal Medicine, University of Occupational and Environmental Health, Kitakyushu, Fukuoka, Japan; 4 Sato Clinic, Tokyo, Japan; 5 Graduate School of Health Innovation, Kanagawa University of Human Services, Kawasaki, Kanagawa Japan; 6 Division of Rheumatology, Department of Internal Medicine, Showa University School of Medicine, Shinagawa-ku, Tokyo, Japan; 7 Department of Healthcare Epidemiology, Kyoto University Graduate School of Medicine and Public Health, Kyoto, Japan; 8 Center for Innovative Research for Communities and Clinical Excellence, Fukushima Medical University, Fukushima, Japan; 9 Research Administration Center, Showa University, Tokyo, Japan; 10 Department of Nephrology, Rheumatology, Endocrinology and Metabolism, Okayama University Graduate School of Medicine, Dentistry and Pharmaceutical Sciences, Okayama, Japan; 11 Department of Clinical Epidemiology, Kochi Medical School, Kochi University, Nankoku, Japan; 12 Department of Stem Cell and Immune Regulation, Yokohama City University Graduate School of Medicine, Yokohama, Japan; 13 Department of Medicine (Neurology and Rheumatology), Shinshu University School of Medicine, Matsumoto, Japan; 14 Center for Rheumatic Diseases, Yokohama City University Medical Center, Yokohama, Japan; 15 Department of Rheumatology and Applied Immunology Faculty of Medicine, Saitama Medical University, Saitama, Japan; 16 Department of Immunology and Rheumatology, Advanced Preventive Medical Sciences, Graduate School of Biomedical Sciences, Nagasaki University, Nagasaki, Japan; 17 Department of Rheumatology, Fukushima Medical University School of Medicine, Fukushima, Japan

**Keywords:** Glucocorticoids, Infection, Prospective cohort study, Systemic lupus erythematosus

## Abstract

**Background:**

Infection is a major cause of mortality in patients with systemic lupus erythematosus (SLE). Therefore, minimizing the risk of infection is an important clinical goal to improve the long-term prognosis of SLE patients. Treatment with ≥7.5 mg prednisolone (PSL) or equivalent has been reported to increase the risk of infections. However, it remains unclear whether <7.5 mg PSL or equivalent dose affects the risk of infection in SLE patients. This study evaluated the association between the occurrence of infection in patients with SLE and low-dose glucocorticoid (GC) usage, especially <7.5 mg PSL or equivalent, to explore the GC dose that could reduce infection occurrence.

**Methods:**

This prospective cohort study included patients from the Japanese multicenter registry of patients with SLE (defined as ≥4 American College of Rheumatology 1997 revised criteria) over 20 years of age. The PSL dose was categorized as PSL 0–2.5, 2.6–5.0, 5.1–7.5, and 7.6–15.0 mg. The primary outcome was infection requiring hospitalization. We conducted a multivariable analysis using time-dependent Cox regression analysis to assess the hazard ratio of infection occurrence compared with a dose of 0–2.5 mg PSL or equivalent in the other three PSL dose groups. Based on previous reports and clinical importance, the covariates selected were age, sex, and concurrent use of immunosuppressants with GC. In addition, two sensitivity analyses were conducted.

**Results:**

The mean age of the 509 SLE patients was 46.7 years; 89.0% were female, and 77.2% used multiple immunosuppressants concomitantly. During the observation period, 52 infections requiring hospitalization occurred. The incidence of infection with a PSL dose of 5.0–7.5 mg was significantly higher than that in the PSL 0–2.5 mg group (adjusted hazard ratio: 6.80, 95% confidence interval: 2.17–21.27). The results of the two sensitivity analyses were similar.

**Conclusions:**

Our results suggested that the use of 5.0–7.5 mg PSL or equivalent could pose an infection risk in SLE patients. This finding indicates that PSL dose should be reduced to as low as possible in SLE patients to avoid infection.

**Supplementary Information:**

The online version contains supplementary material available at 10.1186/s13075-022-02869-9.

## Background

Infection is a major cause of mortality in patients with systemic lupus erythematosus (SLE) [1, 2]. Therefore, minimizing the risk of infection is an important clinical goal to improve the prognosis of SLE patients. Previous studies have suggested that the use of prednisolone (PSL) at a dose ≥7.5 mg, high SLE disease activity, intravenous administration of cyclophosphamide, a history of lupus nephritis, decreased white blood cell count, anti-dsDNA IgG levels >20 IU/ml, and decreased complement levels were risk factors for infection in SLE patients [3–6].

Regarding the risk of infection with glucocorticoids (GCs), previous exploratory studies have suggested that daily PSL at a dose higher than 7.5 mg posed a severe infection risk in SLE patients [7–10]. In rheumatoid arthritis patients, an increased infection risk has been reported even at PSL doses ≤5 mg [11]. In addition, a cohort study assessing the risk of hospitalization in patients with rheumatic diseases and COVID-19 found that >5 mg PSL was associated with higher odds for hospitalization [12]. These studies showed that even doses of PSL <7.5 mg could be risk factors for infection and hospitalization in patients with rheumatic diseases. Thus, we hypothesized that a PSL dose <7.5 mg could pose an infection risk in SLE patients, as with other rheumatic diseases.

However, no study to date has assessed whether PSL <7.5 mg daily or equivalent GC dose increases the infection risk in SLE patients. In addition, the lupus low disease activity state (LLDAS) has recently been described as a clinically relevant treatment target in SLE patients, and current PSL or equivalent dose ≤7.5 mg is one of the LLDAS criteria [13]. LLDAS attainment has been reported to decrease organ damage induced by SLE [14]. However, it is unknown whether achieving this target PSL dosage could reduce infection in SLE patients.

If a PSL dose <7.5 mg poses an infection risk in SLE patients, it is necessary to achieve maintenance therapy with a PSL dose lower than the currently recommended dose. This study investigated the relationship between low-dose GC (≤7.5 mg PSL or equivalent) and infection occurrence in SLE patients, using a database of the multicenter registry of SLE patients in Japan.

## Methods

### Aim

This study aimed to investigate the relationship between low-dose GC (≤7.5 mg PSL or equivalent) and infection occurrence in SLE patients, using a database of the multicenter registry of SLE patients in Japan.

### Study design

This study was a prospective cohort study using data from the Lupus Registry of Nationwide Institutions (LUNA), a multicenter database of patients with SLE in Japan.

### Setting

The participating institutions included the rheumatology departments of eight university hospitals and one general hospital. Because this study included data from multiple institutions from various regions of Japan, we were able to minimize selection bias and achieve geographical representativeness. The patients were registered regardless of whether they were outpatients or hospitalized when the registration started.

Data on GC use were collected at each visit, and data regarding other patient characteristics were collected once a year at each hospital. Patient data were collected until the patient’s death, referred to other hospitals for non-medical reasons, such as relocation, or the patient’s decision to withdraw from the registry.

### Participants

Patients were recruited from February 2016 to September 2019. We included SLE patients over 20 years who fulfilled the American College of Rheumatology criteria for SLE classification [15], including patients with SLE complicated with other connective tissue diseases (such as Sjogren’s syndrome). The included patients were followed up for more than 1 year. We excluded patients who received daily PSL >15 mg or equivalent during the study period because this dose is already known to be an infection risk, and our focus was on the relationship between low-dose PSL and infection. Patients with missing data regarding GC, infection, or follow-up were also excluded.

### Variables

We recorded the following from the database: age, sex, SLE disease duration, comorbid diabetes mellitus (defined by a serum HbA1c > 6.5%), biopsy-proven lupus nephritis (class III, IV, and V according to the classification of the International Society of Nephrology and the Renal Pathology Society 2003 [16]), the SLE Disease Activity Index 2000 [17], chronic kidney disease complication (defined as eGFR < 60 ml/min/1.73m^2^), blood serum data (C3, C4, CH50, anti-ds-DNA IgG antibody, and IgG), white blood cell count, concurrent use of immunosuppressants (defined by the use of tacrolimus, cyclosporine, mycophenolate mofetil, azathioprine, or mizoribine), past immunosuppressant use (methylprednisolone pulse therapy, intravenous cyclophosphamide, and rituximab), and hydroxychloroquine (HCQ). We recorded variables at the time of registration, and time-varying variables were also collected once a year when the patients were followed up in the registry.

### Primary exposure

The primary exposure was systemic GC administration. The GC dose was averaged to the daily dose and converted to the PSL dose. The PSL dose was categorized as PSL 0–2.5, 2.6–5.0, 5.1–7.5, and 7.6–15.0 mg, as previously described [18]. Moreover, we considered this categorization to be clinically relevant as it is unknown whether PSL doses <7.5 mg could pose an infection risk, and the LLDAS aims to use a PSL dose <7.5 mg.

### Outcome measure

The primary outcome was infection requiring hospitalization. This outcome was selected as it is clinically relevant for patients; furthermore, infections requiring hospitalization are usually severe and may require changes in the therapy plan. Data on hospitalizations for infection were identified from the records of the hospitals participating in this study. Hospital records were also reviewed to verify whether patients were admitted to any other hospitals not participating in LUNA. Infections were classified as respiratory infection, urinary tract infection, abdominal infection, soft tissue and bone infection, neurological infection, and others. In patients with multiple infections, each infection was considered and evaluated.

### Statistical analysis

Age, SLE Disease Activity Index, blood serum data, and white blood cell count were used as continuous variables. Sex, comorbid diabetes mellitus, lupus nephritis, PSL dose group, concurrent use of multiple immunosuppressants, past use of intravenous immunosuppressive therapy, HCQ use, and infection occurrence were used as binary variables. Summary statistics are presented as mean values with standard deviations and as numbers with proportions.

We conducted a univariable analysis on variables between the infection and non-infection groups. Additionally, we conducted a univariate analysis between the four groups divided by the registered PSL dose groups. Continuous variables were analyzed with the *t*-test and categorical variables were analyzed with the chi-square test.

We conducted a multivariable analysis using time-dependent Cox regression analysis to assess the hazard ratio of infection occurrence with PSL dose, referenced to PSL or equivalent dose of 0–2.5 mg. The covariates selected were age, sex, and concurrent immunosuppressant use because these variables are clinically important for infection occurrence in SLE patients, as previously reported [19, 20]. The covariate data were collected at registration and every other year.

We conducted two sensitivity analyses. First, we conducted a time-dependent Cox regression analysis among the patients who did not receive HCQ, because HCQ has a protective effect against infection in SLE [21]. Second, we conducted a Cox regression analysis for infection risk with total PSL dose in 1 year in each group among the patients whose PSL dose did not change over the course of 1 year. This was because the PSL dose was changed every hospital visit and therefore we were unable to accurately determine the effect of GC after every dose change. We conducted the same analysis as the primary analysis in the sensitivity analyses.

We assumed that data were missing at random, and thus, missing data were handled by using multiple imputations, a method for handling missing data in epidemiological and clinical research, for multivariate analysis. Statistical significance was defined as a two-sided *p*-value < 0.05. All statistical analyses were conducted using STATA 15.1 (Stata Corp LP, College Station, TX, USA).

### Patient and public involvement

Patients and the public were not involved in the design, conduct, and reporting of this research.

## Results

### Study population

**Fig. 1:**
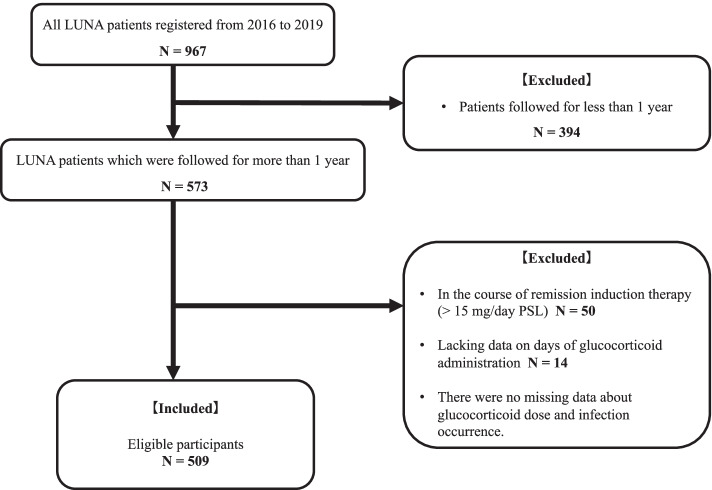
Flowchart of eligible and ineligible participants. A total of 573 LUNA participants were followed up after 1 year, and 394 patients were excluded because of insufficient follow-up duration. The total number of excluded patients was 64 (50 patients who were undergoing remission therapy with PSL >15 mg; 14 patients with missing data on the number of days of glucocorticoid administration). There were no cases lacking data on glucocorticoid dose and infection occurrence. The final number of eligible participants was 509

A total of 966 SLE patients were extracted from the database, and 394 patients were excluded because of insufficient follow-up periods. Sixty-four patients were excluded because they were administered >15 mg PSL or equivalent dose within 1 year (*n* = 50) or had missing data on days of PSL administration (*n* = 14). No patients had missing data about the PSL dose and infection occurrence. Consequently, 509 patients were included in the analysis of the relationship between PSL dose and infection occurrence (Fig. 1).

### Baseline characteristics

Table 1 shows the baseline characteristics of included patients. On average, participants were middle-aged, and most of them were females. These results were similar to those of a previous epidemiological study [22]. The total follow-up time was 946.2 patient-years, and the mean follow-up duration in each patient was 675.3 days. The follow-up times in each group were 939.9 patient-years in the PSL 0–2.5 mg, 939.5 patient-years in the PSL 2.6–5.0 mg, 931.6 patient-years in the PSL 5.1–7.5 mg, and 929.3 patient-years in the PSL 7.6–15.0 mg groups. The infection group was older on average, had a longer follow-up duration, and was administrated methylprednisolone pulse therapy more frequently compared with the non-infection group. The patient characteristics divided by the glucocorticoid dose groups are shown in Table S1.

**Table 1:** Patient characteristics

	Infection group (*N* = 50)	Non-infection group (*N* = 459)	*p*-value	Total patients (*n* = 509)	Missing data
Age (years), mean ± SD	52.3 ± 15.6	46.1 ± 14.6	0.009	46.7 ± 14.8	0
Sex (female), *n* (%)	43 (89%)	410 (86%)	0.63	453 (89.0%)	0
Follow-up days, mean ± SD	802.5 ± 324.7	661.5 ± 342.7	0.006	675.3 ± 343.3	0
Disease duration (year), mean ± SD	11.8 ± 8.8	13.4 ± 10.1	0.304	13.3 ± 10.4	0
Glucocorticoid dose at the time of registration					0
PSL 0–2.5 mg	2 (4.0%)	90 (19.2%)	0.011	92 (18.0%)	
PSL 2.6–5.0mg	16 (32.0%)	160 (35.2%)	0.805	176 (34.6%)	
PSL 5.1–7.5mg	10 (20.0%)	70 (14.8%)	0.506	80 (15.7%)	
PSL 7.6–15.0mg	22 (44.0%)	139 (30.8%)	0.07	151 (29.7%)	
mPSL pulse therapy, *n* (%)	27 (54%)	170 (37%)	0.02	197 (38.7%)	0
Immunosuppressant, *n* (%)	41 (82%)	352 (76.7%)	0.5	393 (77.2%)	0
Cyclophosphamide, *n* (%)	18 (36%)	112 (24.4%)	0.11	130 (25.5%)	0
Tacrolimus, *n* (%)	16 (32%)	148 (32.2%)	1	164 (32.2%)	0
Cyclosporin, *n* (%)	6 (12%)	24 (5.2%)	0.11	30 (5.9%)	0
Mycophenolate mofetil, *n* (%)	7 (14%)	54 (11.8%)	0.81	61 (12.0%)	0
Azathioprine, *n* (%)	8 (16%)	68 (14.8%)	0.99	76 (14.9%)	0
Mizoribine, *n* (%)	4 (8%)	17 (3.7%)	0.28	21 (4.1%)	0
Methotrexate, *n* (%)	1 (2%)	17 (3.7%)	0.83	18 (3.5%)	0
Rituximab, *n* (%)	3 (6%)	6 (1.3%)	0.68	9 (1.8%)	0
Hydroxychloroquine, *n* (%)	9 (18%)	78 (17%)	1	87 (17.1%)	0
WBC (/μL), mean ± SD	6410.8 ± 2561.3	5750.1 ± 2046.2	0.08	5815.1 ± 2108.8	1 (2.0%)
HbA1c > 6.5%	6 (14.3%)	18 (5%)	0.94	24 (4.7%)	105 (20.6%)
Lupus nephritis (class III, IV, V)	9 (18.4%)	118 (27.1%)	0.94	127 (25.0%)	21 (4.1%)
(Biopsy was performed 195/509)					
CKD complication	97 (21.2%)	14 (28.6%)	0.314	111 (21.9%)	2 (0.4%)
Current smoker	6 (12%)	50 (10.9%)	1	118 (23.2%)	28 (5.5%)
SLEDAI score, mean ± SD	5.7 ± 5.0	5.1 ± 4.6	0.51	5.2 ± 4.6	49 (9.6%)
C3 (mg/dl), mean ± SD	89.2 ± 24.5	82.4 ± 21.9	0.07	83.1 ± 22.3	23 (4.5%)
C4 (mg/dl), mean ± SD	18.8 ± 10.2	16.8 ± 8.6	0.19	17.0 ± 8.8	11 (2.1%)
CH50 (U/ml), mean ± SD	36.9 ± 10.0	35.4 ± 11.0	0.35	35.6 ± 10.9	13 (2.6%)
Anti-ds DNA (IU/ml), mean ± SD	16.3 ± 26.3	21.2 ± 37.5	0.26	20.8 ± 36.6	23 (4.5%)
IgG (mg/dl), mean ± SD	1309 ± 490.4	1422.1 ± 476.8	0.16	1411.7 ± 478.6	57 (11.2%)
Pneumococcal vaccination	8 (17.0%)	49 (11.2%)	0.34	57 (11.1%)	23 (4.5%)

*SD* standard deviation, *WBC* white blood cell count, *CKD* chronic kidney disease, *SLEDAI* SLE Disease Activity Index, *Anti-ds DNA* anti-double strand DNA antibody

### Incidence of infection

There were 50 patients with infections, and 52 infection occurrences during the study period (Table 2).

**Table 2:** Infection occurrence during the study period (*n* = 52)

	Infection occurrence	Percentage
Respiratory	25	48.1%
Urinary	12	23.1%
Abdominal	10	19.2%
Soft tissue	3	5.8%
Others	2	

Most infections were respiratory (48.1%), followed by urinary tract infections (23.1%) and abdominal infections (19.2%).

### Associations between PSL dose and infection occurrence

**Fig. 2:**
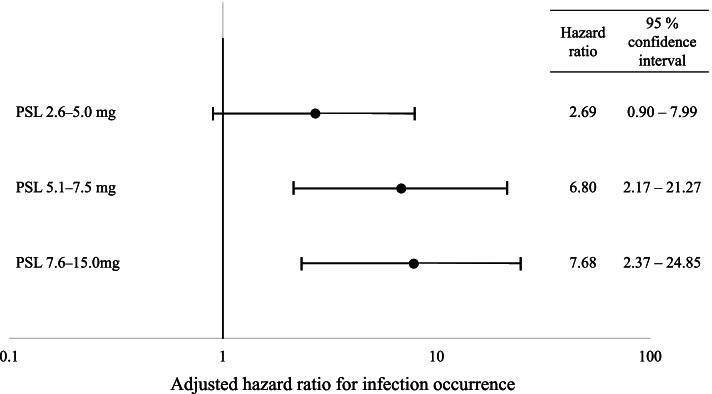
Forest plot of the adjusted hazard ratio of infection occurrence. Age, sex, and concurrent immunosuppressant use were used as covariates

The adjusted hazard ratio of infection occurrence compared with the PSL 0–2.5 mg group was 2.69 (95% CI 0.90–7.99) in the PSL 2.6–5.0 mg group, 6.80 (95% CI 2.17–21.27) in the PSL 5.1–7.5 mg group, and 7.68 (95% CI 2.38–24.85) in the PSL 7.6–15.0 mg group (Fig. 2).

### Sensitivity analysis

The results of the two sensitivity analyses are presented in Tables 3 and 4.

**Table 3:** Hazard ratio for infection occurrence in patients who were not administered hydroxychloroquine, adjusted by age, sex, and immunosuppressant use

Glucocorticoid dose (PSL or equivalent)	Hazard ratio for infection occurrence (95% confidence interval)	
	Unadjusted	Adjusted
PSL 0–2.5 mg	Reference	Reference
PSL 2.6–5.0 mg	2.27 (0.76–6.80)	2.51 (0.83–7.59)
PSL 5.1–7.5 mg	3.81 (1.17–12.43)	5.28 (1.57–17.77)
PSL 7.6–15.0 mg	5.46 (1.72–17.38)	8.56 (2.55–28.72)

**Table 4:** Hazard ratio for infection occurrence in patients with fixed PSL dose for 1 year

Glucocorticoid dose (PSL or equivalent)	Hazard ratio for infection occurrence (95% confidence interval)	
	Unadjusted	Adjusted
PSL 0–2.5 mg	Reference	Reference
PSL 2.6–5.0 mg	6.78 (0.88–52.56)	6.97 (0.89–54.41)
PSL 5.1–7.5 mg	9.15 (1.02–81.99)	9.67 (1.07–87.17)
PSL 7.6–15.0 mg	6.58 (0.68–63.27)	7.53 (0.75–75.54)

First, the time-dependent Cox regression analysis among 508 patients who were not administered HCQ revealed that PSL >5.0 mg increased the risk of infection compared with PSL 0–2.5 mg (Table 3). This result of time-dependent Cox regression analysis was consistent with that of the main analysis. Second, the Cox regression analysis among 329 patients whose PSL dose did not change in 1 year showed that a PSL dose of 5.1–7.5 mg increased the risk of infection compared with a dose of 0–2.5 mg (Table 4). However, the risk of infection was not significantly higher in patients treated with a PSL dose of 7.6–15.0 mg.

## Discussion

This study investigated the association between a low GC dose and infection occurrence in patients with SLE, using a multicenter registry database in Japan. The results suggested that, compared with a PSL dose of 0–2.5 mg, administration of PSL 5.0–7.5 mg was associated with an increased infection risk in SLE patients. Our findings indicated that reducing the PSL doses to ≤ 5.0 mg may be necessary to decrease infection occurrence.

In this study, the hazard ratio of infection occurrence in SLE patients administered 5.0–7.5 mg PSL was 6.80 when compared to those administered with 0–2.5 mg PSL. Hence, even 5.0–7.5 mg PSL dose could pose an infection risk in SLE patients. Previous exploratory research showed that a PSL dose >7.5 mg increased the risk of infection in SLE patients [10], and this was consistent with our observations.

Unlike previous reports which did not find any increased risk of infection with lower-dose PSL, our observations may be explained by two main reasons. First, this may have been due to differences in statistical analysis. Our study was analyzed using a time-dependent Cox regression analysis, whereas previous studies assessed the effect of GC on infection based on the highest GC dose administered in the past and the GC dose at the time of study registration [8, 9]. Because the GC dose is often changed at each hospital visit, previous studies did not assess the effect of the current GC dose on infection correctly. In contrast, our study collected data on the GC dose at each hospital visit, and we assessed the effect of GC on infection by time-dependent Cox regression analysis. Therefore, ours was a more accurate evaluation of the relationship between infection occurrence and GC dose. Second, previous studies were exploratory and retrospective [8, 9], while our study was exploratory and prospective. Therefore, we were able to estimate confounding factors and had fewer missing data. Thus, our results more accurately assessed the relationship between infection and GC dose.

Several mechanisms can explain the increased risk for infection with low-dose GC. GCs have various effects on inflammatory and immunologically mediated processes, such as impeding the access of neutrophils and monocytes to inflammatory sites and also cause lymphocytopenia [23]. These effects proved the infection occurrence. However, in an in vitro experiment, only middle- to high-dose GC doses impaired granulocyte phagocytosis [24]. Nevertheless, the results of our own and another study [10] indicate that low-dose GC can predispose to infection. Thus, low-dose GC appears to have an effect on immunity; however, further studies are required to elucidate the underlying mechanism.

Our study had several strengths. First, our study was conducted using a multicenter registry. Therefore, our study results can be generalized to most patients with SLE and current clinical practice. Second, we adjusted for confounding factors affecting infection occurrence based on previous reports. Therefore, our study allowed for a precise evaluation of the correlation between GC and infection risk in patients with SLE.

This study also had several limitations. First, we could not assess each immunosuppressant. Nonetheless, it was reported that infection risk did not differ among SLE patients treated with mycophenolate mofetil, azathioprine, and cyclophosphamide [25]. Hence, the lack of a classification of immunosuppressants is unlikely to have had marked effects. Second, we could not evaluate GC accumulation. Accumulation of the GC dose has been reported to be related to infection risk [26]. Nevertheless, the recent GC dose has a greater impact on infection than the accumulated GC dose in rheumatoid arthritis patients [27]. Thus, this limitation is unlikely to have had a marked effect on our findings. Third, we could not adjust for the effect of HCQ on infection occurrence. HCQ has been reported to have a protective role against infection [21]. Thus, our analysis may underestimate the relationship between GC and infection occurrence. However, the rate of HCQ use in our registry was low because HCQ has only recently become available for use in Japan. In addition, we conducted a sensitivity analysis to assess the association of low PSL dose and infection occurrence in patients with SLE who were not administered HCQ, and this sensitivity analysis yielded a similar result to the main analysis. Fourth, pneumococcal vaccination was not assessed as a confounding factor. It has been reported that pneumococcal infections are frequent and severe in patients with SLE [10, 28]. Pneumococcal vaccination efficacy in SLE patients has been established [29]. However, we could not adjust for pneumococcal vaccination status because of the patient number. Nevertheless, the number of vaccinated patients was only 11.1%, and thus, the influence was likely to be minimal. Fifth, most of the patients in this registry were Japanese and were followed in eight university hospitals and one general hospital. Therefore, it might not be possible to generalize our study results to non-Japanese patients and to those followed in private practice. Sixth, we were unable to adjust the main analysis by disease duration. Longer disease duration can be an infection risk. However, there was no significant difference in disease duration between the infection and non-infection groups. Therefore, we concluded that the survivor bias due to the differences in disease duration was low in this study.

## Conclusions

Our time-dependent Cox regression analysis of data from the SLE registry in Japan suggested that even low-dose GC, 5.0–7.5 mg PSL or equivalent, could increase the infection risk in SLE patients. This finding indicated that PSL dose should be reduced to as low as possible in SLE patients to avoid infection.

## Supplementary Information

**Additional file 1: Table S1**: Patient characteristics divided by baseline glucocorticoid dose.

## Data Availability

The datasets used and analyzed during the current study are available from the corresponding author on reasonable request.

## Abbreviations

PSL: Prednisolone
SLE: Systemic lupus erythematosus
GC: Glucocorticoid
LLDAS: Lupus low disease activity state
LUNA: Lupus Registry of Nationwide Institutions
HCQ: Hydroxychloroquine
