# Supplementary material for: Association of low-dose glucocorticoid use and infection occurrence in systemic lupus erythematosus patients: a prospective cohort study
Source: Arthritis Res Ther. 2022 Jul 28;24:179. doi: 10.1186/s13075-022-02869-9 (PMC9330647; doi:10.1186/s13075-022-02869-9)
Supplement: Supplementary file 1 — Additional file 1: Table S1: Patient characteristics divided by baseline glucocorticoid dose. [file 13075_2022_2869_MOESM1_ESM.docx]

Table S1: Patient characteristics divided by baseline glucocorticoid dose

| Baseline PSL group | PSL 0 - 2.5mg (N = 92) | PSL 2.6 – 5.0mg (N = 176) | PSL 5.1 - 7.5mg (N = 80) | PSL 7.6mg – 15.0 mg (N = 161) | P-value |
| --- | --- | --- | --- | --- | --- |
| Infection occurrence (%) | 2 (2.2 %) | 16 (9.1 %) | 10 (12.5 %) | 22 (13.7%) | 0.023 |
| Age (years), mean ± SD | 52.0 ± 16.0 | 49.6 ± 14.7 | 44.7 ± 13.8 | 41.6 ± 12.9 | <0.001 |
| Sex (female), n (%) | 80 (87.0 %) | 159 (90.3 %) | 71 (88.8%) | 143 (88.8%) | 0.867 |
| Disease duration (year), mean ± SD | 15.8 ± 12.1 | 14.3 ±  10.4 | 10.9 ±  7.8 | 11.95 ± 10.1 | 0.005 |
| Follow-up days, mean ± SD | 596.7 ± 327.1 | 695.1 ± 343.5 | 722.7 ± 349.7 | 675.1 ± 344.7 | 0.074 |
| mPSL pulse therapy, n (%) | 25 (27.2 %) | 64 (36.4 %) | 38 (47.5 %) | 70 (43.5%) | 0.021 |
| Immunosuppressant, n (%) | 60 (65.2 %) | 133 (75.6%) | 66 (82.5%) | 134 (83.2%) | 0.006 |
| Cyclophosphamide, n (%) | 15 (16.3 %) | 42 (23.9 %) | 28 (35.0 %) | 45 (28.0 %) | 0.034 |
| Tacrolimus, n (%) | 23 (25.0%) | 48 (27.3 %) | 30 (37.5 %) | 63 (39.1 %) | 0.033 |
| Cyclosporin, n (%) | 1 (1.1 %) | 11 (6.2 %) | 4 (5.0 %) | 14 (8.7 %) | 0.099 |
| Mycophenolate mofetil, n (%) | 8 (8.7 %) | 12 (6.8 %) | 11 (13.8 %) | 30 (18.6 %) | 0.006 |
| Azathioprine, n (%) | 8 (8.7 %) | 24 (13.6 %) | 14 (17.5 %) | 30 (18.6 %) | 0.158 |
| Mizoribine, n (%) | 2 (2.2 %) | 7 (4.0 %) | 7 (8.8%) | 5 (3.1%) | 0.13 |
| Methotrexate, n (%) | 1 (1.1 %) | 6 (3.4 %) | 5 (6.2 %) | 6 (3.7 %) | 0.338 |
| Rituximab, n (%) | 0 (0 %) | 2 (1.1 %) | 2 (2.5 %) | 5 (3.1 %) | 0.265 |
| Hydroxychloroquine, n (%) | 12 (13.0 %) | 25 (14.2 %) | 13 (16.2 %) | 37 (23.0%) | 0.108 |
| WBC (/μL), mean ± SD | 5199.9 ± 1519.1 | 5866.5 ± 1996.9 | 5960.5 ± 2327.6 | 6038.5 ± 2342.2 | 0.017 |
| HbA1c > 6.5% | 0 (0 %) | 8 (5.9 %) | 5 (8.3 %) | 11 (8.1 %) | 0.096 |
| Lupus nephritis (Class Ⅲ, Ⅳ, Ⅴ)  (Biopsy was performed 195/509) | 21 (24.1 %) | 34 (20.6 %) | 27 (34.6 %) | 45 (29.0 %) | 0.095 |
| CKD complication | 18 (19.6 %) | 45 (25.7 %) | 17 (21.2 %) | 31 (19.4 %) | 0.494 |
| Current smoker | 10 (10.9 %) | 16 (9.1 %) | 8 (10.0 %) | 22 (13.7 %) | 0.592 |
| SLEDAI score, mean ± SD | 3.8 ± 3.6 | 4.8 ± 4.9 | 5.2 ± 4.9 | 6.4 ± 4.6 | < 0.001 |
| C3 (mg/dL), mean ± SD | 83.8 ± 19.9 | 86.6 ± 21.7 | 79.8 ± 18.4 | 80.4 ± 25.3 | 0.036 |
| C4 (mg/dl), mean ± SD | 18.0 ± 8.9 | 17.6 ± 8.6 | 16.3 ± 8.3 | 16.0 ± 9.1 | 0.219 |
| CH50 (U/ml), mean ± SD | 36.8 ± 9.4 | 37.7 ± 11.4 | 33.7 ± 9.8 | 33.5 ± 11.4 | 0.001 |
| Anti-ds DNA (IU/ml), mean ± SD | 15.8 ± 27.2 | 19.5 ± 41.1 | 24.2 ± 32.0 | 23.2 ± 37.8 | 0.374 |
| IgG (mg/dl), mean ± SD | 1595.1 ± 500.8 | 1451.6 ± 528.2 | 1348.4 ±395.2 | 1295.5 ± 409.9 | <0.001 |
| Pneumococcal vaccination | 16 (18.4 %) | 27 (16.0 %) | 7 (9.3 %) | 7 (4.5 %) | 0.002 |
